# Immunohistochemical panel to characterize canine prostate carcinomas according to aberrant p63 expression

**DOI:** 10.1371/journal.pone.0199173

**Published:** 2018-06-12

**Authors:** Carlos Eduardo Fonseca-Alves, Priscila Emiko Kobayashi, Luis Gabriel Rivera Calderón, Sérgio Luis Felisbino, Jaqueline de Carvalho Rinaldi, Sandra Aparecida Drigo, Silvia Regina Rogatto, Renée Laufer-Amorim

**Affiliations:** 1 Department of Veterinary Clinic, School of Veterinary Medicine and Animal Science, São Paulo State University–UNESP, Botucatu, SP, Brazil; 2 Department of Morphology, Instituto de Biociências, São Paulo State University–UNESP, Botucatu, SP, Brazil; 3 Department of Clinical Genetics, Vejle Hospital and Institute of Regional Health Research, University of Southern Denmark, Vejle, Denamark; Duke University School of Medicine, UNITED STATES

## Abstract

An unusual variant of prostate adenocarcinoma (PC) expressing nuclear p63 in secretory cells instead of the typical basal expression has been reported in men. Nevertheless, the biological behavior and clinical significance of this phenomenon is unknown. In dogs, this unusual PC subtype has not been described. In this study, p63 immunoexpression was investigated in 90 canine PCs and 20 normal prostate tissues (NT). The p63 expression pattern in luminal or basal cells was confirmed in a selected group of 26 PCs and 20 NT by immunohistochemistry and/or Western blotting assays. Eleven canine PC samples aberrantly expressing p63 (p63+) in secretory cells were compared with 15 p63 negative (p63-) cases in the context of several molecular markers (high molecular weight cytokeratin-HMWC, CK8/18, CK5, AR, PSA, chromogranin, NKX3.1, PTEN, AKT and C-MYC). P63+ samples were positive for CK5, HMWC and CK8/18 and negative for PSA, NKX3.1, PTEN and chromogranin. Five p63+ PCs were negative for AR, and the remaining six samples had low AR expression. In contrast, p63- PC showed AR and PSA positive expression in all 15 samples. Only five p63- PCs were positive for CK5. Both p63+ and p63- PC samples showed higher cytoplasmic AKT expression and nuclear C-MYC staining in comparison with normal tissues. Metastatic (N = 12) and non-metastatic (N = 14) PCs showed similar immunoexpression for all markers tested. In contrast to human PC, canine PC aberrantly expressing p63 showed higher expression levels of HMWC and CK5 and lower levels of NKX3.1. Canine p63+ PC is a very rare PC group showing a distinct phenotype compared to typical canine PC, including AR and PSA negative expression. Although in a limited number of cases, p63 expression was not associated with metastasis in canine PC, and cytoplasmic p63 expression was observed in animals with shorter survival time, similar to human PC cases.

## Introduction

Canine prostate cancer (PC) represents 13% of all prostatic disorders, and its aggressive behavior is associated with a high metastasis rate and poor prognosis [1,2]. Canine PC has been proposed as a comparative oncology model, which is useful in better understanding human PC [1]. Nevertheless, differences between canine and human prostate glands have been reported [2,3]. Human normal prostatic tissues present a continuous basal cell layer positive for p63. However, during carcinogenesis, the basal cell layer is lost, and typical human PC lacks p63 positive cells [4,5]. Normal luminal human prostatic cells are negative for p63, uroplakin III and high molecular weight cytokeratin (HMWC) and present positivity for prostatic specific antigen (PSA), low weight cytokeratin (cytokeratin 8 and 18), NKX3.1 and androgen receptor (AR) expression [6,7]. In normal canine prostatic tissues, a discontinuous basal cell layer and fewer basal cells [3,8,9] have been reported. Due to the discontinuity of the basal cell layer in normal canine prostate, basal cell markers have not been used for PC diagnosis in veterinary medicine [8].

The most common type, acinar prostate adenocarcinoma, presents a luminal phenotype, which expresses AR and low weight cytokeratin (cytokeratin 8 and 18), while the basal cell markers (p63, CK5 and HMWC) are not expressed [5]. In contrast, a rare group of unusual prostatic adenocarcinomas presents nuclear p63 expression with non-basal distribution [5,10–12]. Secretory luminal cells from unusual PC variants were characterized by p63 positivity and the absence of CK5 and HMWC expression [13]. Recently, Tan et al [5] proposed that PC aberrantly expressing p63 is an entity molecularly distinct from the usual-type PC.

In veterinary medicine, three previous studies have evaluated the expression of p63 in canine prostatic tissues [8,9,14]. To the best of our knowledge, no report on canine PC expressing p63 in secretory cells has been conducted. Despite that dogs are an important model of human prostatic diseases, the relevance of p63 expression in dogs is poorly explored.

In the present study, we analyzed p63 expression in a large number of canine PCs. Afterward, we compared the findings with an extensive immunohistochemical panel composed of luminal/basal markers and proteins commonly evaluated in typical human prostate carcinomas.

## Materials and methods

### Ethics statement

This study was performed in accordance with the National and International Recommendations for the Care and Use of Animals. All procedures were performed under the approval of the Animal Ethics Committee from the Faculty of Veterinary Medicine and Animal Science, UNESP, Botucatu, SP, Brazil (Protocol 107/2015).

### Patients and sample selection

Ninety canine formalin fixed paraffin-embedded (FFPE) PC samples collected from 2011 to 2015 were retrieved from the archives of the Veterinary Pathology Service, Veterinary School, UNESP, SP-Brazil. Fresh frozen tumor tissues were available in 26 of these 90 FFPE samples (S1 Fig). Frozen tumor samples were obtained during prostatectomy, biopsy or necropsy. The interval between death and necropsy was less than 30 minutes.

Twenty FFPE normal prostatic gland tissues (NT) and 10 paired fresh frozen NT samples were also available for the experiments (S1 Fig). Non-malignant samples with no inflammation (normal samples) were selected according to Fonseca-Alves et al. [15]. Briefly, normal samples were composed of glandular columnar cells with tissue stroma composed of muscle fibers, collagen and stromal cells. Normal samples showed no inflammatory infiltrates or any histological alterations. Normal tissues were collected during necropsy from animals that died from causes not related to prostatic disease. All samples (90 PCs and 20 NT) were from intact dogs.

Medical records were reviewed to obtain clinical information, treatment protocols, treatment responses and outcomes (S1 Table). Radical prostatectomy was performed in three animals with non-metastatic disease. From these 26 patients, 10 received metronomic chemotherapy, four only piroxicam, five were not treated and from the remaining four cases, follow up was lost. Metronomic chemotherapy was provided according to Fonseca-Alves et al. [16]. Unfortunately, outcome information was unavailable in nine of the 26 patients.

To exclude urothelial origin, uroplakin III immunostaining (UPIII) was performed in all tumors (N = 90) (S2 Fig) according to Lai et al. [17]. Forty-seven of the 90 tumors were UPIII positive and 43 were negative. Based on the amount of tissue in the paraffin block and its paired frozen sample, 26 UPIII negative PCs (43/90) were selected for further analysis (S1 Fig). These 26 PC samples (3 from prostatectomy, 12 biopsies and 11 necropsies) were evaluated for p63 protein expression by immunohistochemistry (Autostainer Classic (Dako Cytomation, Carpinteria, USA). This analysis revealed 11 p63 luminal positive cells (p63+ PC) and 15 showed exclusively p63+ basal cells (p63- PC).

Metastatic disease was found in 12 of these 26 cases (UPIII negative tumors). We selected seven metastatic samples from p63+ PC and five from p63- PC. The selection criteria were based on the amount of tissue available for the proposed analysis and the presence of at least one metastasis per anatomic site (6 bone, 3 lungs, 2 livers and 1 intestine metastasis) (data detailed in S1 Table). Metastatic samples were investigated for p63, CK5 and HMWC antibodies due to the limited amount of tissues.

### Histopathological analysis

Histopathological diagnosis was performed according to the human World Health Organization (WHO) classification of Tumors of the Urinary System and Male Genital Organs [18] and adapted to canine PC [17]. Gleason-like score was determined according to Palmieri and Grieco [19].

### Protein expression analysis by immunohistochemistry

The FFEP tissue sections were deparaffinized and rehydrated, and antigen unmasking was performed using citrate buffer (pH 6.0) for 30 seconds in a pressure cooker (Pascal®; Dako, Carpinteria, CA, USA). The tissue sections were treated with 3% hydrogen peroxide in methanol for 20 min and washed in Tris-buffered saline. The primary antibodies and their respective dilutions are shown in S2 Table. A peroxidase-conjugated polymer system (Envision, Dako, Carpinteria, CA, USA) was utilized as a secondary antibody. The slides were incubated with chromogen 3´-diaminobenzidine tetrahydrochloride (DAB, Dako, Carpinteria, CA, USA) for 5 min, and counterstained with Harris hematoxylin. Negative controls were performed for all antibodies using a rabbit immunoglobulin fraction (Dako, Carpinteria, CA, USA) and a mouse universal negative control (Dako, Carpinteria, CA, USA), according to the manufacturer’s instructions. Normal canine prostatic tissue was used as the positive control for NKX3.1 and PSA, the canine uterine cervix was used as the positive control for AKT and C-MYC, the canine stomach was used as the positive control for PTEN, normal testes were used as the positive control for AR, and normal mammary glands were used as the positive control for CK8/18, CK5, p63, HMWC, CD44 and CD24 expression. Normal pancreatic tissue was used as the positive control for chromogranin, and canine normal lymph nodes were used as the positive control for Ki67 expression and bladder tissue for positive UPIII staining. The tissues selected as positive controls followed the Protein Atlas recommendation (https://www.proteinatlas.org/).

The cross reactivity with canine tissue was provided by the manufacturer’s datasheet for NKX3.1, C-MYC, PSA, CK5, Ki67, AKT and chromogranin. Canine tissues show cross reactivity of AR, PTEN [20] and CK8/18 antibodies [21]. CD44, CD24 and UPIII antibodies were previously validated in canine tissues [17,22]. In this study, the cross reactivity for HMWC and p63 were tested for Western blotting.

Protein expression of p63, HMWC, Ki67, CK8/18, CK5, CD44, CD24, PTEN, AKT, C-MYC, chromogranin, NKX3.1, AR and PSA were evaluated according to Wang et al. [23]. The analysis was performed in tumor samples grouped according to scores: score 0 (absence of staining), 1 (5–30% of positive cells), 2 (30–50% of positive cells), 3 (50–75% of positive cells) and 4 (>75% of positive tumor cells).

### P63-CK8/18 double immunostaining

The double immunohistochemical analysis for p63-CK8/18 was performed to classify tumors as aberrantly expressing p63 [24]. Briefly, the tissue samples were deparaffinized, and antigen retrieval was performed using citric acid solution (pH 6.0) in a pressure chamber (Pascal^®^, Dako, Carpinteria, USA). The endogenous peroxidase activity was blocked with 3% H_2_O_2_ diluted in methanol. Unspecific protein binding was blocked with a commercial protein block solution (Pascal^®^, Dako, Carpinteria, USA). The primary antibody, anti-CK8/18 (Clone 5D3, Leica Biosystems, Wetzlar, Germany) at 1:600 dilution, was incubated overnight at 4°C. A polymer was used as a secondary antibody (Envision, Dako, Carpinteria, USA). After a one-hour incubation with the secondary antibody, 3 3'-diaminobenzidine chromogen (DAB^®^, Dako, Carpinteria, USA) was applied, producing the brown color. The second primary antibody (anti-p63; clone 4A4; Dako, Carpinteria, USA) was applied at 1:50 dilution, overnight at 4°C. For the second staining, the same polymer system (Envision, Dako, Carpinteria, USA) was applied for one hour, followed by the red chromogen (3-amino-9-ethylcarbazole chromogen—AEC) (Dako, Carpinteria, USA). Harris hematoxylin was used for counterstaining and the slides were mounted in an aqueous medium (Dako, Carpinteria, USA).

Tumor cells positive for both p63 and luminal cell markers (CK8+/18+) were considered as having aberrant p63 expression [5].

### Western blotting

Ten normal prostatic and 11 p63+ PC fresh tissues were histopathologically re-evaluated to confirm the diagnosis or to exclude proliferative inflammatory atrophy (PIA), benign prostatic hyperplasia (BPH) or necrosis. Three of the 10 normal samples were excluded due to the presence of surrounding PIA or BPH lesions. Four of the 11 p63 positive PC samples were excluded due to extensive necrosis or BPH area surrounding the neoplastic cells. Western blotting (WB) was performed for 14 tissues (7 normal prostatic tissues and 7 p63+ PC) to confirm the cross-reactivity of p63 and HMWC antibodies with canine tissues.

The prostate samples were homogenized (Polytron homogenizer, Kinematica, Lucerne, Switzerland) for 30 s at 4°C in 50 mM Tris–HCl buffer pH 7.5, 0.25% Triton X-100 and EDTA. After centrifugation, protein was extracted from the supernatant and quantified as described by Bradford [25]. Equal amounts of protein (70 μg) from each sample were heated at 95°C for 5 min in sample loading buffer, subjected to SDS–PAGE or electrophoresis under reducing conditions and then transferred to nitrocellulose membranes (Sigma Chemical Co., St. Louis, MO). The blots were blocked with 3% bovine serum albumin in TBS-T (10 mM Tris–HCl pH 7.5, 150 mM NaCl, and 0.1% Tween-20) for 1 h and probed overnight with p63 and HMWC antibodies. Goat anti-β-actin antibody (1:1,000; sc-1615, Santa Cruz Biotechnology, Santa Cruz, CA, USA) was used as a loading control. After incubation with the corresponding horseradish peroxidase-conjugated secondary antibody, the blots were visualized via chemiluminescence (Amersham ECL Select Western Blotting Detection Reagent, GE Healthcare). Protein bands were quantified by densitometry and expressed as the integrated optical density (IOD).

### Statistical analysis

The sample size was calculated with the G-Power program (G-power®, Brunsbüttel, Germany) using a type 1 error of 5%, and power of 80%. The chi-squared exact test was used to compare the protein immunoexpression between canine PC samples (p63+ and p63-) and normal samples. Survival curves were generated using the Kaplan-Meier method, and statistical significance was determined using a log-rank test. Overall survival was defined as the period (in months) between the date of surgery and death caused by the disease. For statistical purpose, primary tumors were also grouped in metastatic tumors *versus* non-metastatic tumors independently of p63 expression. T test was used to analyze the Western blotting data. *P*<0.05 was considered significant for all analyses. Statistical analysis was performed using GraphPad Prism 5 (GraphPad Software Inc., La Jolla, CA) software.

## Results

### Histological pattern

Among the 11 p63+ PC cases, the cribriform architecture was found in five PCs, four showed small acinar and two mixed patterns (cribriform and small acinar) (S1 Table). The Gleason-like score varied from 6 to 10 (2 cases: 6, 2 cases: 8 and 7 cases: 10) (S1 Table). Six of the 15 p63- PCs presented the small acinar pattern, five had a solid pattern and four presented cribriform pattern. The Gleason-like score was 10 in nine cases and 6 in the remaining six p63- tumors (S1 Table).

### Immunohistochemical expression of basal cell markers

**Fig 1:**
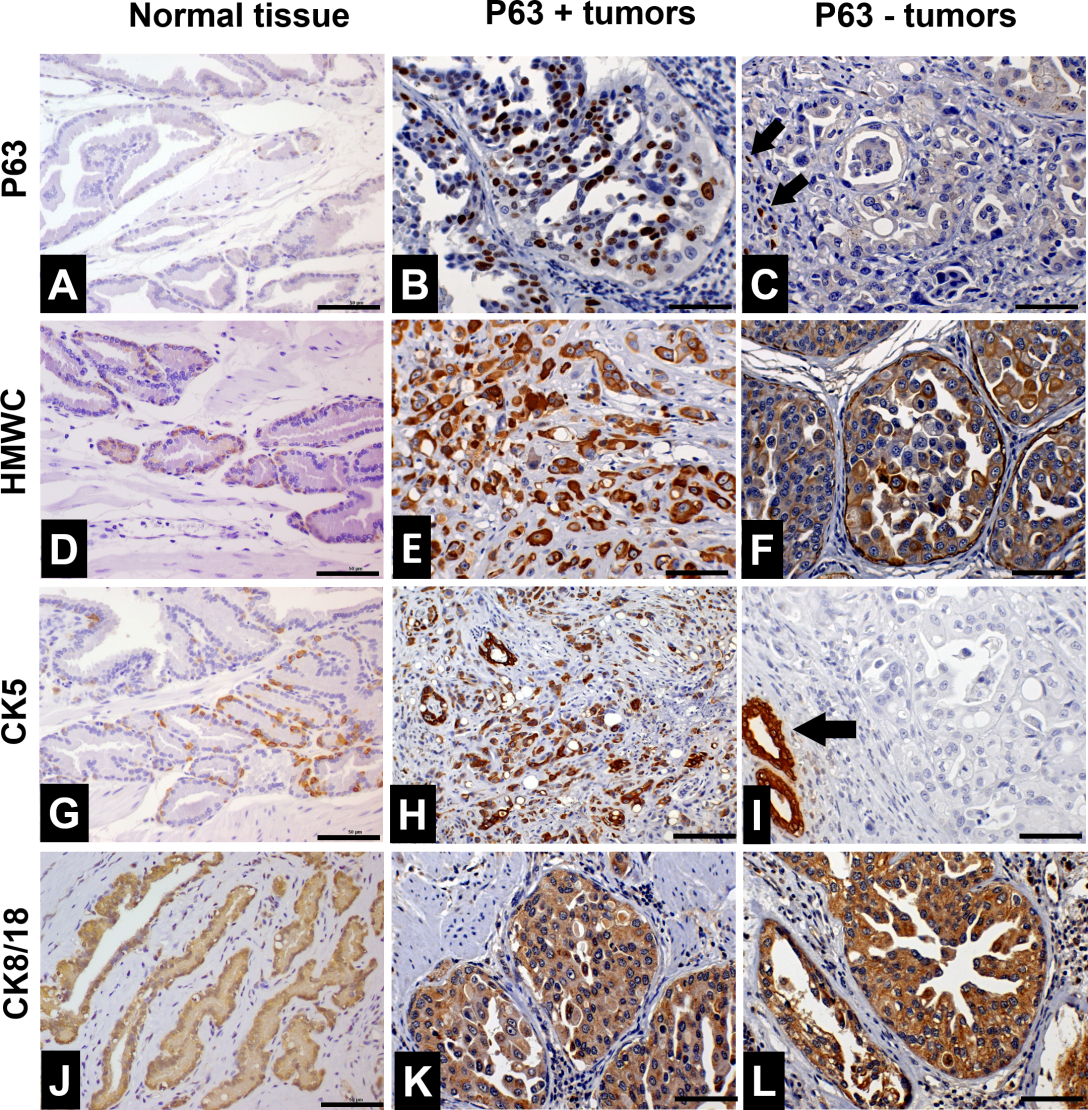
Characterization of basal cell markers in normal prostate samples, canine p63+ PC and canine p63- PC. (A) p63 immunohistochemical expression in a normal prostate gland. Positive basal cells were observed in a discontinuous basal cell layer. (B) p63+ tumor showing diffuse nuclear p63 expression by neoplastic cells. (C) p63- tumor presenting no p63 expression (blue staining). Few non-neoplastic basal cells with nuclear p63 expression (internal control–black arrows) were observed. (D) Immunohistochemical staining of high molecular weight cytokeratin (HMWC) in a normal prostate gland. Positive staining was observed in few basal cells (brown staining—white arrows) establishing a discontinuous basal cell layer. (E and F) Diffuse HMWC expression in p63+ and p63- tumors, respectively. (G) CK5 immunohistochemical expression in canine normal samples. There was a discontinuous basal cell layer with few CK5 positive cells (brown staining). (H) p63+ tumor showing diffuse CK5 expression (brown staining) in all neoplastic cells. (I) p63 tumor showing no CK5 expression by neoplastic cells (blue staining). An atrophic prostatic gland (black arrow) was observed presenting diffuse CK5 expression (internal control). (J) CK8/18 expression in normal prostate. Positive cytoplasmic expression was observed in the epithelial luminal cells (brown staining). p63+ (K) and p63 (L) tumors presented diffuse cytoplasmic staining in all neoplastic cells (brown staining). The negative staining can be observed as a blue signal.

The normal prostate glands (all 20 cases) presented nuclear p63 expression only in basal cells, forming a discontinuous layer (Fig 1A) (Table 1). The p63+ tumors (all 11 cases) presented nuclear p63 positive expression in luminal and basal cells (Fig 1B). Four p63+ PC samples presented cytoplasmic p63 expression and the remaining seven cases had only nuclear immunostaining. Furthermore, p63- tumors showed no p63 nuclear expression in luminal cells (15/15). In p63- tumor samples, only sporadic p63 positive cells distributed randomly were observed (Fig 1C).

**Table 1:** Immunohistochemical panel evaluated in normal canine prostate tissues and neoplastic prostate cells according to p63 expression.

	Basal markers*			Luminal markers			Proliferative marker	Markers reported in typical human PC**					
Identification	p63	CK5	HMWC	CK8/ CK18	AR	PSA	Ki67	PTEN	AKT	C-MYC	NKX3.1	CD44+ /CD24-	Chromogranin
*p63-positive*													
1	3	4	4	3	0	1	3	0	4	4	0	2	0
2	3	2	4	4	1	0	3	1	4	4	0	1	0
3	2	2	4	3	1	1	2	1	4	4	0	2	0
4	2	4	4	4	1	0	3	0	4	4	0	1	0
5	2	2	4	2	0	0	2	0	4	4	0	1	0
6	2	2	4	3	1	0	3	1	4	4	0	2	0
7	3	2	4	3	1	1	2	0	4	4	0	1	0
8	2	2	4	2	0	0	3	1	4	4	0	2	0
9	2	4	4	3	1	1	3	0	4	4	0	2	0
10	3	3	4	3	0	1	2	0	4	4	0	1	0
11	3	2	4	3	2	0	3	1	4	4	1	3	0
*p63-negative*													
12	0	0	2	3	1	1	2	0	3	4	1	0	0
13	0	0	3	4	2	2	3	2	3	4	0	2	0
14	0	0	4	3	1	1	4	0	4	4	0	0	0
15	0	1	2	4	1	3	3	1	3	4	0	0	0
16	0	0	4	3	1	1	4	0	4	4	0	2	0
17	0	1	3	4	1	1	3	1	3	4	1	0	0
18	0	0	4	3	1	2	4	0	4	4	0	1	0
19	0	0	2	4	1	1	3	2	3	4	0	0	0
20	0	0	3	3	1	3	4	0	4	4	0	1	0
21	0	0	2	2	1	1	3	1	3	4	1	0	0
22	0	0	3	4	2	1	4	2	3	3	2	1	0
23	0	1	3	4	1	2	3	1	3	4	1	0	0
24	0	1	2	3	0	4	3	3	2	3	3	0	0
25	0	0	3	4	1	2	3	2	3	4	0	2	0
26	0	1	3	3	1	2	2	1	1	4	2	1	0
*Normal prostate*							3						
1	0	0	0	4	4	4	0	3	1	1	4	0	
2	0	0	0	4	4	4	0	4	1	1	4	0	0
3	0	0	0	4	4	3	0	3	0	1	4	0	0
4	0	0	0	4	4	4	0	4	1	1	4	0	0
5	0	0	0	4	4	3	0	3	0	1	4	0	0
6	0	0	0	4	4	4	0	4	1	1	4	0	0
7	0	0	0	4	4	4	0	3	0	2	4	0	0
8	0	0	0	4	4	4	0	4	1	1	4	0	0
9	0	0	0	4	4	4	0	3	0	2	4	0	0
10	0	0	0	4	4	4	0	4	1	1	4	0	0
11	0	0	0	4	4	3	0	4	1	0	4	0	0
12	0	0	0	4	3	3	0	4	0	0	4	0	0
13	0	0	0	4	4	3	0	4	0	0	4	0	0
14	0	0	0	4	4	3	0	4	0	1	4	0	0
15	0	0	0	4	4	4	0	4	0	0	4	0	0
16	0	0	0	4	4	4	0	4	0	0	4	0	0
17	0	0	0	4	4	4	0	4	0	2	4	0	0
18	0	0	0	4	4	4	0	3	0	1	4	0	0
19	0	0	0	3	4	3	0	4	0	0	4	0	0
20	0	0	0	4	4	4	0	4	1	0	4	0	0

PC: prostate cancer; HMWC: high molecular weight cytokeratin; CK: cytokeratin. Score 0: absence of expression; Score 1: (5–30% of positive cells); score 2: (30–50% of positive cells); score 3: (50–75% of positive cells) and score 4: (>75% of positive tumor cells).

*Basal cell marker expression in prostatic luminal cells

**All samples showed absence of chromogranin expression.

HMWC positive expression was found only in basal cells, forming a discontinuous basal cell layer (Fig 1D). P63+ tumor cells showed higher expression of HMWC compared to normal tissue samples (Fig 1E) (Table 1), while p63- tumor cells presented HMWC cytoplasmic expression (Fig 1F). Interestingly, a continuous layer of basal cells was observed in both p63+ and p63- tumors, which was not detected in normal tissues (S3 Fig).

The normal prostatic samples (all 20) presented no CK5 expression in normal luminal cells, but the basal cells were positive, forming a discontinuous basal cell layer (Fig 1G). CK5 positive expression was observed in p63+ tumor cells (Fig 1H). In contrast, few p63- tumor samples (5 of 15) had CK5 expression (Fig 1I). P63+ tumors had higher CK5 expression levels compared to p63- tumors (P = 0.001).

### Immunohistochemical Expression of Prostatic Luminal Markers

Among the luminal markers used in human prostatic pathology, we investigated CK8/18, PSA, NKX3.1 and AR protein expression. All normal samples (20/20) had positive CK8/18 expression in luminal cells (score 4) (Fig 1J). The CK8/18 positivity was detected in both, p63+ (11 cases) (Fig 1K) and p63- (15 cases) (Fig 1L) tumor samples (scores 3 or 4) (Table 1).

Luminal cells were positive for PSA in all 20 normal prostatic samples. Five of the 11 p63+ tumors presented PSA expression in luminal cells (score 1), while all p63- PCs (15 cases) were positive for PSA (Table 1).

**Fig 2:**
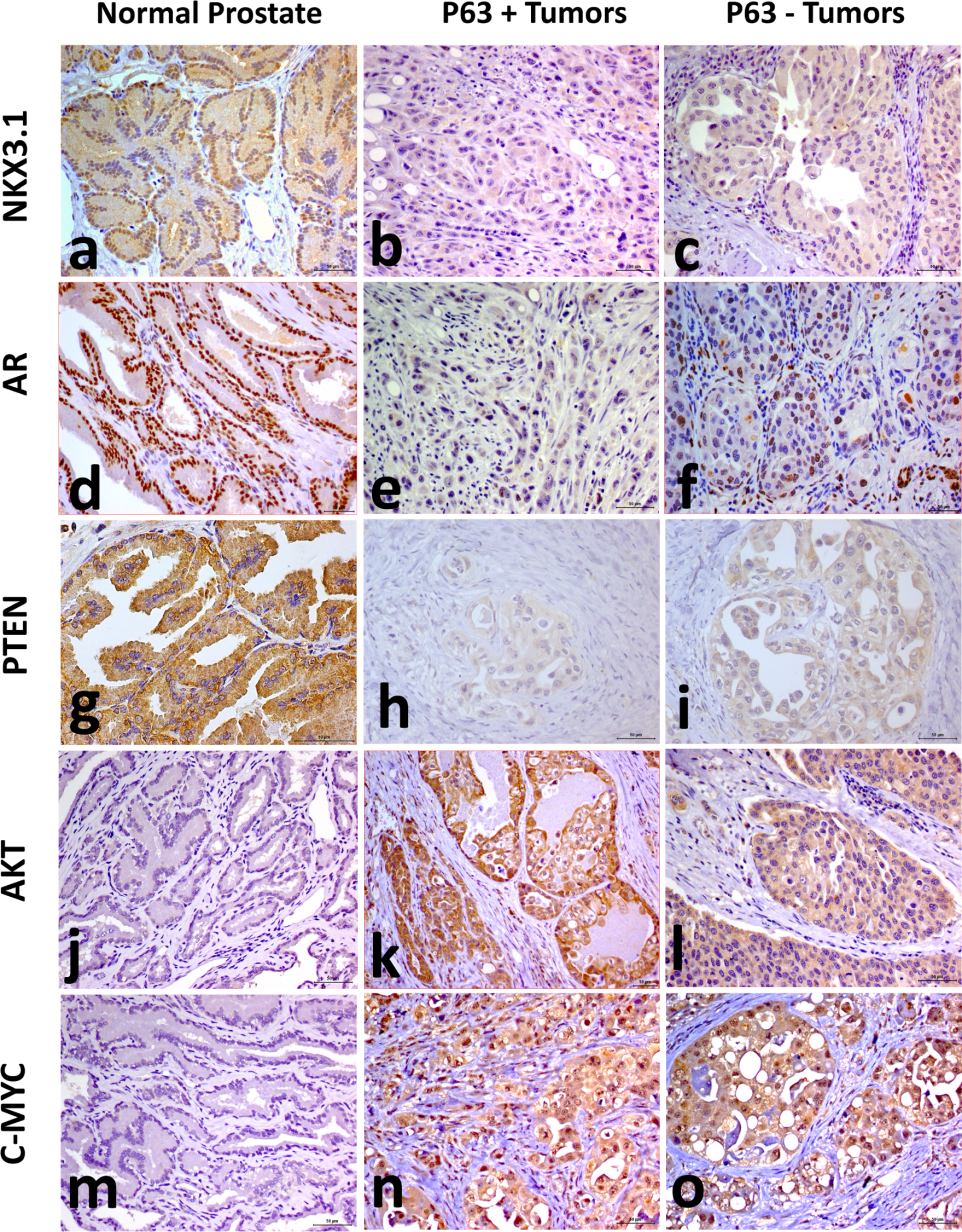
Immunohistochemical staining for NKX3.1, AR, PTEN, AKT AND C-MYC in canine prostate cancer. (A) Normal prostatic tissue presented strong expression of NKX3.1. All p63+ and p63- PCs were negative for NKX3.1 staining (B and C). D: Strong expression of AR was observed in normal prostatic tissue. (E and F) Few neoplastic and basal cells were positive for AR in p63+ PC and p63- PC. (G) Normal prostatic tissue presented strong expression of PTEN. p63+ PC (H) and p63- PC (L) showed low PTEN expression. (J) Normal prostatic tissue showed AKT negative PTEN expression (blue staining). (K and L) Strong expression in p63+ and P63- PC was noted. (M) Normal prostatic tissue showed negative C-MYC expression, and higher expression was noted in both p63+ (N) and p63- (O) tumors.

NKX3.1 expression was found in luminal cells from all normal samples (20/20) and no basal or stromal cells expressed NKX3.1 (Fig 2A). Only one (1/11) p63+ PC and seven P63- PCs had NKX3.1 expression (score 1) (Fig 2B and 2C) (Table 1). There was decreased NKX3.1 expression in p63+ and p63- PCs compared to normal samples (P = 0.0001).

AR was expressed in all normal prostate samples (20/20) in luminal cells (Fig 2D) and few stromal cells. Basal cells were negative for AR. Seven p63+ (Fig 2E) and 14 p63- PCs (Fig 2F) (4/11) had positive AR expression (score 1 or 2) (Table 1). There was decreased AR expression in p63+ (P = 0.0001) and p63- (P = 0.0001) PCs compared to normal samples.

### Immunohistochemical expression of PTEN, AKT, C-MYC, CD44, CD24 and chromogranin

PTEN expression was detected in luminal cells from all normal prostate samples (Fig 2G) (Table 1). Four of the 11 p63+ and 10 of the 15 p63- tumors showed PTEN positive expression (score 1). Decreased PTEN expression levels were observed in p63+ (P = 0.001) (Fig 2H) and p63- tumors (P = 0.001) (Fig 2I) compared to normal samples.

Eight normal samples (8/20) showed AKT positive expression (score 1) in luminal cells. Fewer basal/stromal cells had AKT positive expression (Fig 2J). AKT positive expression (score 4) was observed in all tumor samples, p63+ and p63- (Table 1) (Fig 2K and 2L). However, significant increased levels of AKT expression were detected in tumors (p63+ and p63-: P = 0.001) compared to normal samples.

Luminal cells from 13 normal samples (13/20) presented C-MYC expression (score 1 or 2), while few basal/stromal cells were positive for C-MYC (Fig 2M). All tumor samples showed significant increased expression levels of C-MYC (score 4) compared to normal samples (P = 0.001 in both, p63+ and p63- tumors) (Fig 2N and 2O).

CD44 and CD24 immunoexpression levels were investigated in sequential sections of the FFPE tissue blocks. The area under investigation was demarcated, and the presence of CD44+/CD24- negative cells was investigated in all prostatic tissues. Normal luminal prostatic cells were negative for both markers and few basal/luminal cells had the CD44+/CD24- phenotype in normal samples. The PC samples (p63+ and p63-) presented CD44+/CD24- cells.

Lack of chromogranin expression was observed in luminal and basal cells from normal and tumor samples. However, sporadic stromal cells from normal samples showed chromogranin expression.

### Immunoexpression of p63, CK5 and HMWC in metastatic samples

Twelve animals presented metastasis, seven being p63+ PCs and five p63- PCs. The metastasis from p63+ PCs presented positive staining for p63 and HMWC (S4 Fig). Interestingly, the metastasis from PC p63+ had no CK5 expression, while metastasis from p63- PCs lacked expression of CK5, p63 and HMWC. The primary tumors (p63+ and p63-) and their respective metastases presented similar expression levels of HMWC.

### Double immunostaining

**Fig 3:**
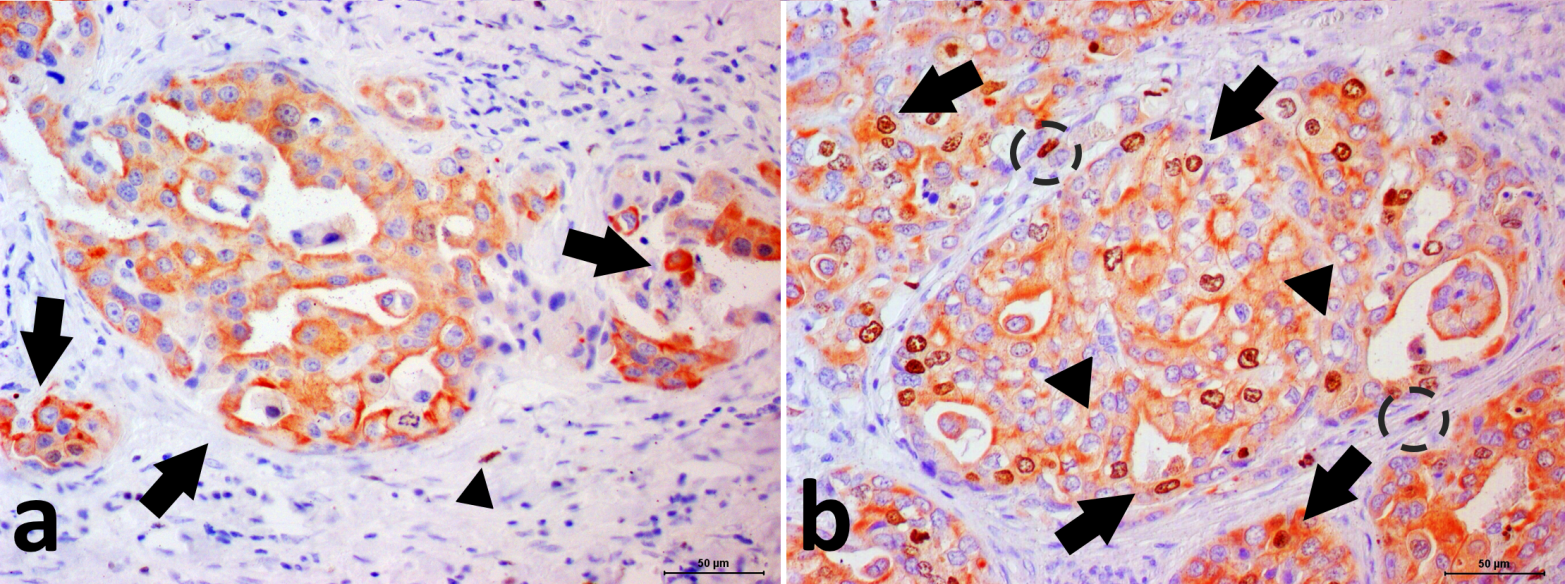
Photomicrograph showing the canine prostate cancer. **Double immunohistochemical staining for p63 and CK8/18.** (A) PC with p63-/CK8/18+ phenotype. It is possible to note cytoplasmic CK8/18 expression (red staining—arrow) in neoplastic cells and nuclear negative p63 staining in neoplastic cells. There is a p63+/CK8/18- basal cell (arrowhead) (internal control). Negative signal can be observed as the blue color. (B) PC with p63+/CK8/18+ phenotype. Note the neoplastic doubled stained cells (arrows). It is also observed p63-/CK8/18+ cells (arrowhead) and p63+/CK8/18- basal cells (doted circle).

Double immunostaining in prostate cancer cells (cytoplasm labeled in red indicates CK8/18 positivity and nuclei labeled in brown indicate p63 positivity) confirmed the aberrant expression of the p63 phenotype. Normal samples had no double positivity for p63 and CK8/CK18 (Fig 3).

### Western blot

**Fig 4:**
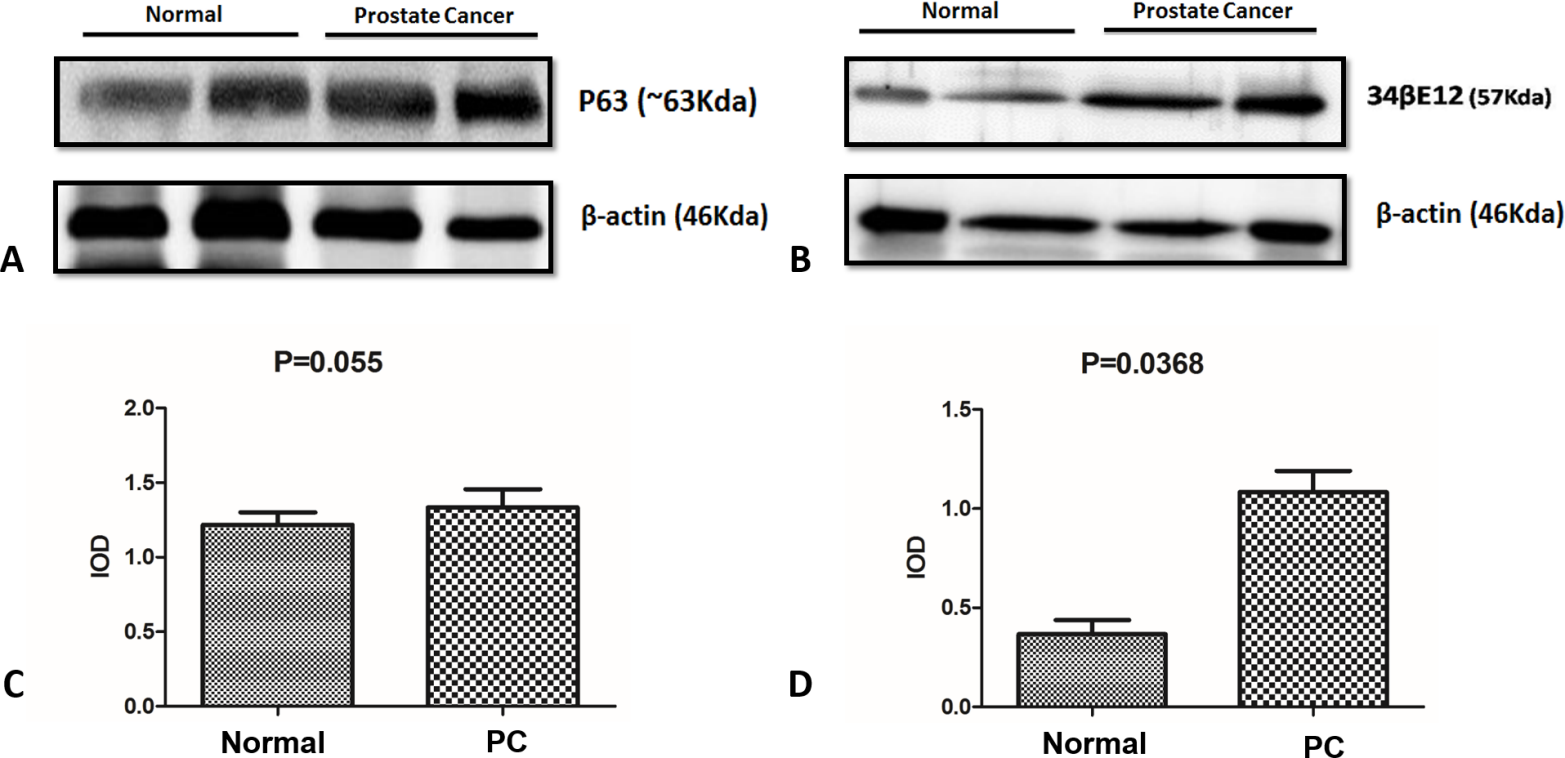
Western blotting analysis. There was no difference in p63 expression in tumor samples compared to normal samples (A and C) and there was higher expression of HMWC in tumor samples compared with normal samples (B and D).

The Western blot analysis performed with normal and p63+ PC samples using the p63 and HMWC antibodies revealed specific bands at 63 kDa and 57 kDa, respectively (Fig 4). These results show cross reactivity of these antibodies with canine prostatic tissue.

## Discussion

A rare unusual subtype of canine PC aberrantly expressing p63 in secretory luminal cells was described. Although in a limited number of PC cases, the protein profiling revealed differences compared with the human counterpart. Canine PC has been considered a model for human prostatic diseases. However, the particularities of these species should be evaluated to standardize dogs as a spontaneous model of human disease. In our study, 12.2% (11/90) of all tumors were p63+, suggesting the rarity of this tumor group, which is similar to those described in humans [5]. P63 immunohistochemistry revealed higher p63 expression in p63+ PC compared to normal samples. On the other hand, Western blotting showed no significant difference (P = 0.055). This discrepancy can be explained by the number of samples analyzed for each technique. We had more samples analyzed by immunohistochemistry (11) compared to Western blotting (7).

We found p63 positive expression in luminal cells in the p63+ PC group. We confirmed this expression by double immunostaining (CK8/18+ p63+). Then, we called this group as aberrantly expressing p63 (p63+). Tan et al. [5] described an intermediate phenotype (p63+ and CK8/18+) of neoplastic prostatic cells, similar to our cases. Moreover, we reported negative PSA expression in 45.5% (5/11) of p63+ PC samples, and all tumors were positive for both luminal (CK8/18) and basal (CK5) cell markers, corroborating a mixed luminal and basal phenotype. In normal prostate gland, UPIII stained the plasma membrane of superficial epithelial cells of the urethra (S5 Fig). Ductal and secretory luminal cells do not show UPIII positive staining.

Interestingly, three cases presented p63 nuclear and cytoplasmic expression in neoplastic cells. These patients experienced a shorter survival time (49 days) compared to the whole group (147.1 days), and two of these dogs developed metastatic disease. Similar to our results, Romanucci et al. [9] reported three of 11 canine PCs with cytoplasmic p63 expression, which was associated with Hsp60, Hsp72 and Hsp7 expression. Further studies should be addressed to investigate the association between poor outcome and p63 cytoplasmic expression in canine PC. In human PC, cytoplasmic p63 expression was correlated with increased mortality [12]. Although no statistically significant, the proliferative index based on the Ki67 expression levels revealed differences in the comparison between normal samples and PC p63 positive and negative cases.

The prostate epithelium contains luminal, basal and rare neuroendocrine cells, and prostate cancer may originate from one of these cell types. Here, the neuroendocrine origin of these tumors was refuted since no chromogranin staining was detected in neoplastic samples (both p63-positive and p63-negative PC).

Diffuse HMWC expression was observed in both p63+ and p63- PC. Akter et al. [21] showed CK5-positivity in 13 of 20 canine PC samples, which represents a higher frequency than we reported. However, the authors did not evaluate the p63 protein expression in tumor samples. According to the authors, 25% of the samples were CK5 positive, and 20% were CK14 positive. The positive expression of these basal cell proteins was consistent in different histological PC types, and the role of these cells should be considered in canine carcinogenesis [21]. In p63+ human prostate cancer, Tan et al. [5] reported no expression of HMWC or CK5 and luminal expression of CK18. Overall, these data highlighted differences in the immunoexpression pattern compared with the human PC data, which were negative for HMWC and CK5 and positive for CK8/18. A plausible explanation is that p63+ canine PC is derived from luminal cells (CK8/18) that are dedifferentiating and thus gaining basal cell marker expression (HMWC and CK5). Alternatively, these tumors could arise from basal cells, which are differentiating into luminal cells.

All metastasis from p63+ primary PCs showed p63 and HMCW immunoexpression. The metastatic samples were CK5 negative, while the primary tumors were positive, which could be related to the differentiation degree of the metastatic cells. Previous studies have indicated that the epithelial to mesenchymal transition is observed in canine PC and that epithelial markers in metastatic cells are lost [16,26]. Since CK5 is a basal cytokeratin and basal cells lose CK5 expression during differentiation to the luminal phenotype, we believe that the metastatic cells lose CK5 during the process of invasion and do not re-express at the metastatic site. Previously, Akter et al. [5] reported one canine PC sample (CK14, CK8/18, CK5 and AR) with neoplastic emboli showing diffuse expression of CK5. However, the metastasis of this unique case was not evaluated.

In our study, both p63+ and p63- PCs presented negative AR immunostaining. Of note, all samples were obtained from intact dogs, suggesting that this cohort consisted of hormone independent disease. In humans, AR-negative PC is refractory to hormone therapy and has been associated with a more aggressive outcome [27]. In metastatic samples, a significant number of samples were AR negative and CD44+/CD24-, compared to non-metastatic cases. Schroeder et al. [28] showed a correlation between androgen receptor loss and the stem cell-like phenotype in human PC. Furthermore, CD44 was reported as a promising cancer stem cell (CSC) marker in canine PC [29]. However, in our set of tumors (both p63+ and p63-) no correlation was observed between loss of AR expression and the CSC phenotype.

*PTEN* is a tumor suppressor gene widely studied in human prostatic pathology due its role in tumor development [30,31]. *PTEN* loss has been associated with phosphatidylinositol-3,4,5-triphosphate (PIP3) and *AKT* activation, which is related to apoptosis, cellular proliferation and tumorigenesis [30]. Using a mouse model, the conditional ablation of *PTEN* promoted basal-to-luminal differentiation and invasive PC [31]. In our study, both tumor groups showed decreased PTEN expression compared to normal samples, and no difference was found between p63+ and p63- PCs. Thus, our results indicated low expression levels of PTEN in canine PC, independent of p63 status.

Our PC cases showed loss of nuclear PTEN staining and an accumulation of cytoplasmic AKT, suggesting continuous activation of the PI3K/AKT pathway. *PTEN* genomic loss (leading to PI3K/AKT pathway activation) and 8q amplification (including the *MYC* gene) are the most frequent genetic alterations in human prostate cancer (~ 30%) [32]. *MYC* and *AKT* are arguably the most prevalent driver oncogenes in prostate cancer. Our results suggest that C-MYC overexpression occurs in addition to PTEN loss and AKT activation. Although there was no association with p63 expression, our results showed a role for these proteins in canine prostate cancer. We expected to find a loss of PTEN expression in canine p63+ compared to p63- tumors and AKT overexpression in p63+ PCs. Although no differences were detected in PTEN expression, AKT overexpression in p63+ PCs compared to p63- PCs was observed. These two proteins are involved in the same signaling pathway [32]; however, AKT overexpression in p63+ PC is likely independent of PTEN expression.

Cross regulation between NKX3.1 and C-MYC was reported in human prostate tissues [33]. A previous study of our group described a negative correlation between NKX3.1 and C-MYC expression in canine prostate, indicating a relation between both proteins in canine PC [8,34]. In this study, low NKX3.1 expression levels and increased C-MYC expression confirmed our previous data regarding the involvement of these proteins in canine prostate carcinogenesis.

## Conclusions

A specific subset of canine p63+ prostatic carcinomas is reported. These tumors are uncommon in dogs and humans, but canine prostatic carcinomas that aberrantly express p63 have a different phenotype, such as positive expression of CK5 and high molecular weight cytokeratin, negative expression of PSA, NKX3.1 and AR. These results support that canine p63+ prostate cancer is a distinct entity in comparison with the human counterpart. However, dogs with cytoplasmic p63 expression presented shorter survival time, similar to human PC cases.

## Supporting information

S1 Fig Exclusion and inclusion samples criteria. Graphic representation illustrating the samples inclusion/exclusion criteria for each technique.
(DOCX)

S2 Fig Uroplakin III staining. Uroplakin III staining in canine tissues. A: Normal bladder tissue positive for Uroplakin III. B and C: p63+ and p63- tumors respectively, showing no Uroplakin III staining.
(DOCX)

S3 Fig High molecular weight cytokeratin. Canine prostate cancer with a solid pattern showing cytoplasmic expression of high molecular weight cytokeratin (HMWC) and a continuous basal cell layer showing remarkable membranous staining for HMWC. Adjacent normal prostatic tissue was observed showing no expression of HMWC in the epithelial cell cytoplasm and basal cell layer (black arrow).
(DOCX)

S4 Fig p63 expression in lymph node metastasis. p63 immunostaining in lymph node metastasis of canine prostate cancer with aberrant p63 expression. p63-positive epithelial cells were observed invading the lymph node parenchyma (A). B: p63 nuclear immunostaining in neoplastic cells in the same section at higher magnification (20x).
(DOCX)

S5 Fig UPIII expression in canine normal prostate. Uroplakin III (UPIII) staining in canine normal prostatic tissue. UPIII positive cells were found only in the superficial cells of the prostatic urethra (insert).
(DOCX)

S1 Table Clinical and histological data. Clinical and histological findings of prostate cancer from 26 dogs.
(DOCX)

S2 Table Primary antibodies details. Primary antibodies used for immunohistochemical analysis.
(DOCX)
