# Supplementary material for: Immunohistochemical panel to characterize canine prostate carcinomas according to aberrant p63 expression
Source: PLoS One. 2018 Jun 12;13(6):e0199173. doi: 10.1371/journal.pone.0199173 (PMC5997330; doi:10.1371/journal.pone.0199173)

S1 Fig. Graphic representation illustrating the samples inclusion/exclusion criteria for each technique.


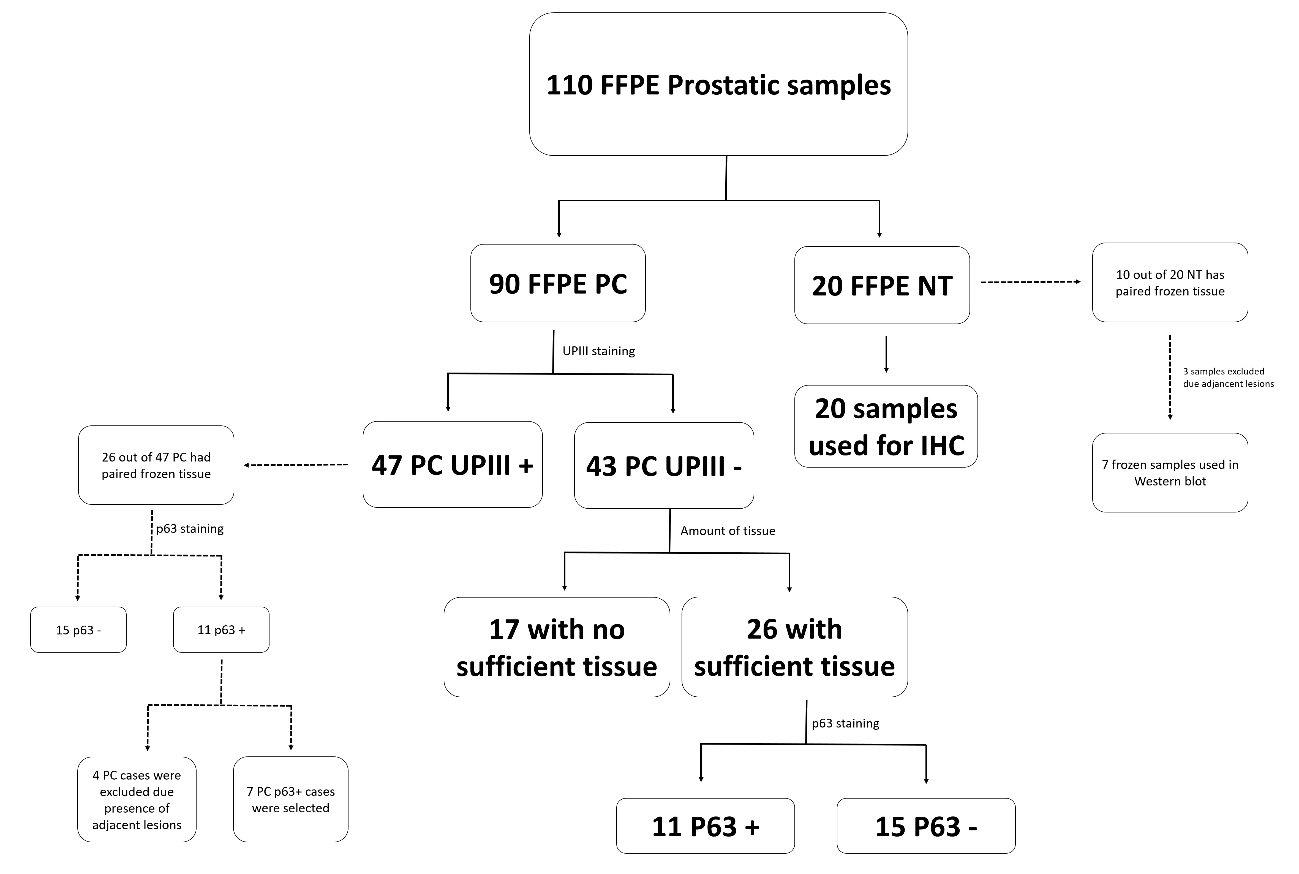

Supplement: S1 Fig — Graphic representation illustrating the samples inclusion/exclusion criteria for each technique. (DOCX) [file pone.0199173.s001.docx]
