# Supplementary material for: Immunohistochemical panel to characterize canine prostate carcinomas according to aberrant p63 expression
Source: PLoS One. 2018 Jun 12;13(6):e0199173. doi: 10.1371/journal.pone.0199173 (PMC5997330; doi:10.1371/journal.pone.0199173)

S2 Fig. Uroplakin III staining in canine tissues. A: Normal bladder tissue positive for Uroplakin III. B and C: p63+ and p63- tumors respectively, showing no Uroplakin III staining


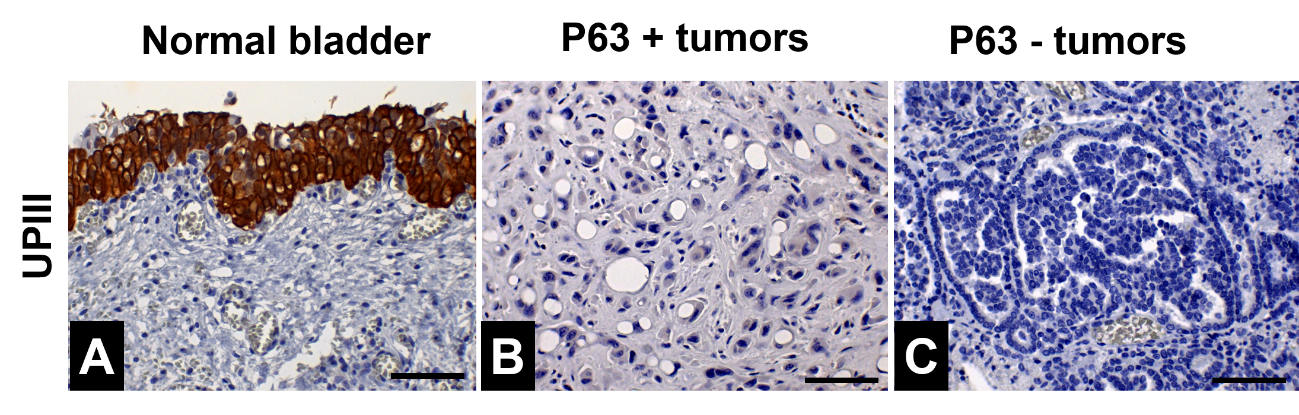

Supplement: S2 Fig — Uroplakin III staining in canine tissues. A: Normal bladder tissue positive for Uroplakin III. B and C: p63+ and p63- tumors respectively, showing no Uroplakin III staining. (DOCX) [file pone.0199173.s002.docx]
