# Supplementary material for: Immunohistochemical panel to characterize canine prostate carcinomas according to aberrant p63 expression
Source: PLoS One. 2018 Jun 12;13(6):e0199173. doi: 10.1371/journal.pone.0199173 (PMC5997330; doi:10.1371/journal.pone.0199173)

S3 Fig. Canine prostate cancer with a solid pattern showing cytoplasmic expression of high molecular weight cytokeratin (HMWC) and a continuous basal cell layer showing remarkable membranous staining for HMWC. Adjacent normal prostatic tissue was observed showing no expression of HMWC in the epithelial cell cytoplasm and basal cell layer (black arrow).


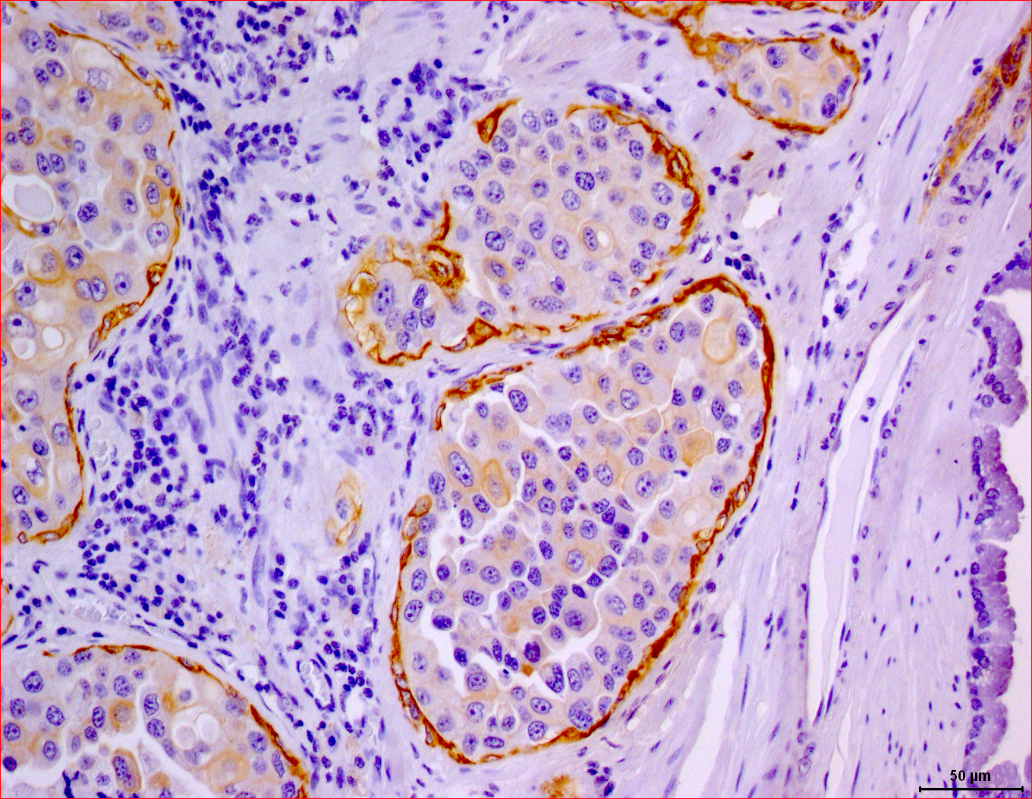

Supplement: S3 Fig — Canine prostate cancer with a solid pattern showing cytoplasmic expression of high molecular weight cytokeratin (HMWC) and a continuous basal cell layer showing remarkable membranous staining for HMWC. Adjacent normal prostatic tissue was observed showing no expression of HMWC in the epithelial cell cytoplasm and basal cell layer (black arrow). (DOCX) [file pone.0199173.s003.docx]
