# Supplementary material for: Immunohistochemical panel to characterize canine prostate carcinomas according to aberrant p63 expression
Source: PLoS One. 2018 Jun 12;13(6):e0199173. doi: 10.1371/journal.pone.0199173 (PMC5997330; doi:10.1371/journal.pone.0199173)

S4 Fig. p63 immunostaining in lymph node metastasis of canine prostate cancer with aberrant p63 expression. p63-positive epithelial cells were observed invading the lymph node parenchyma (A). B: p63 nuclear immunostaining in neoplastic cells in the same section at higher magnification (20x).


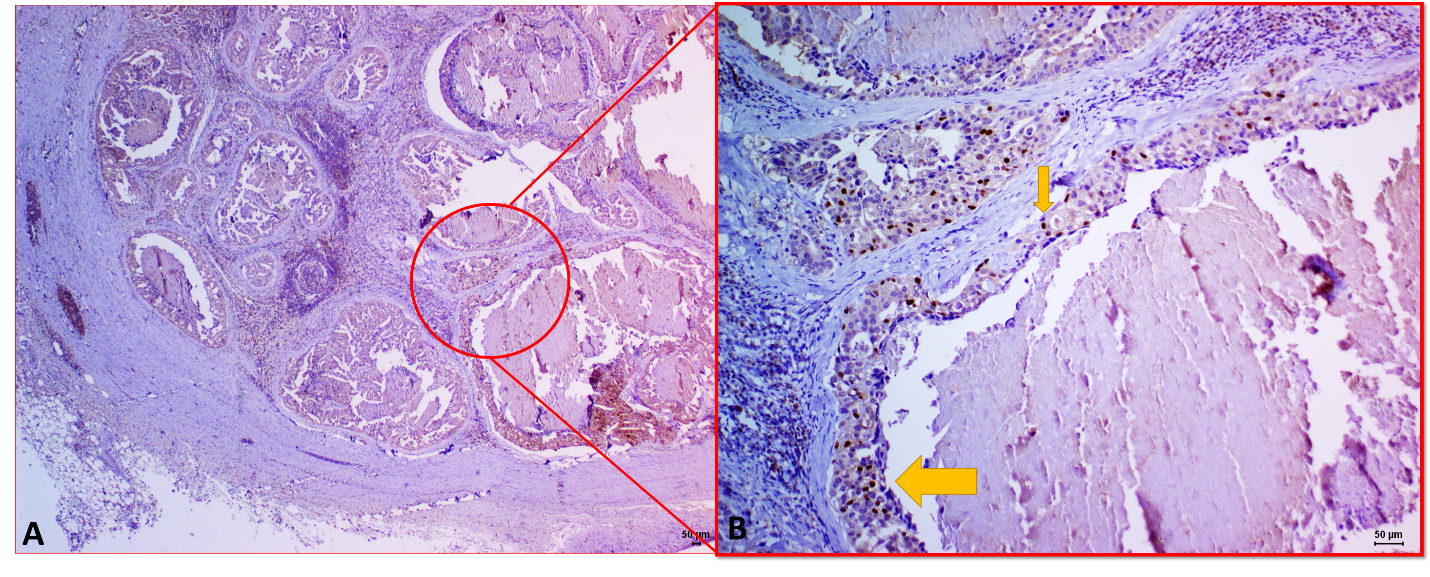

Supplement: S4 Fig — p63 immunostaining in lymph node metastasis of canine prostate cancer with aberrant p63 expression. p63-positive epithelial cells were observed invading the lymph node parenchyma (A). B: p63 nuclear immunostaining in neoplastic cells in the same section at higher magnification (20x). (DOCX) [file pone.0199173.s004.docx]
