# Supplementary material for: Immunohistochemical panel to characterize canine prostate carcinomas according to aberrant p63 expression
Source: PLoS One. 2018 Jun 12;13(6):e0199173. doi: 10.1371/journal.pone.0199173 (PMC5997330; doi:10.1371/journal.pone.0199173)
Supplement: S5 Fig — Uroplakin III (UPIII) staining in canine normal prostatic tissue. UPIII positive cells were found only in the superficial cells of the prostatic urethra (insert). (DOCX) [file pone.0199173.s005.docx]

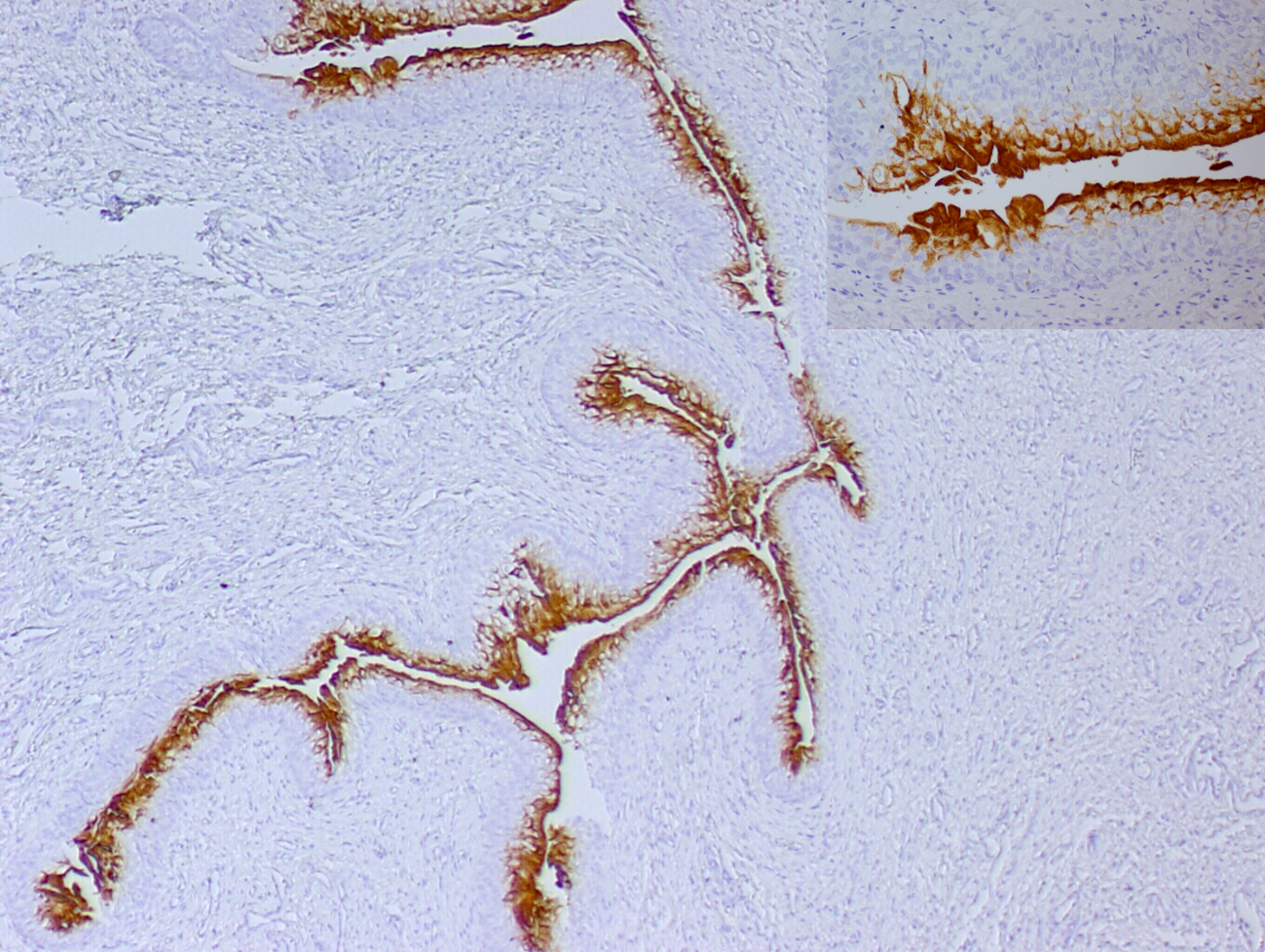
S5 Fig. Uroplakin III (UPIII) staining in canine normal prostatic tissue. UPIII positive cells were found only in the superficial cells of the prostatic urethra (insert).
