# Supplementary material for: Immunohistochemical panel to characterize canine prostate carcinomas according to aberrant p63 expression
Source: PLoS One. 2018 Jun 12;13(6):e0199173. doi: 10.1371/journal.pone.0199173 (PMC5997330; doi:10.1371/journal.pone.0199173)
Supplement: S1 Table — Clinical and histological findings of prostate cancer from 26 dogs. (DOCX) [file pone.0199173.s006.docx]

**S1 Table.** Clinical and histological findings of prostate cancer from 26 dogs.

| **Prostatic Cancer Case** | **Breed** | **Age (years)** | **Metastasis** | **Histological Pattern** | **Gleason-like score*** | **Treatment** | **Outcome (days)** |
| --- | --- | --- | --- | --- | --- | --- | --- |
|  |  |  |  |  |  |  |  |
| *p63 aberrantly expressed* | |  |  |  |  |  |  |
| 1 | Boxer | 14 | Lung, Bone and Liver | Cribriform | 10 | Piroxicam | 90 |
| 2 | Teckel | 11 | No | Cribriform | 10 | N/A | N/A |
| 3 | Boxer | 12 | Bone, Lung | Cribriform | 10 | LDMT | 278 |
| 4 | MBD | 15 | Bone, Lung | Cribriform | 10 | LDMT | 423 |
| 5 | MBD | 13 | Bones, Intestine, Lung | Small acinar + Cribriform | 8 | N/T | 12 |
| 6 | German Shepherd | 10 | No | Small acinar + Cribriform | 8 | N/T | N/A |
| 7 | Poodle | 8 | No | Small acinar | 6 | RP | 45 |
| 8 | American Cocker Spaniel | 10 | No | Small acinar | 10 | RP | 32 |
| 9 | American Pitbull Terrier | 10 | Bones, Lung | Small acinar | 6 | Piroxicam | N/A |
| 10 | MBD | 14 | Lung, intestine | Small acinar | 10 | N/A | N/A |
| 11 | MBD | 9 | Bone | Cribriform | 10 | LDMT | 150 |
| *p63 negative* | |  |  |  |  |  |  |
| 12 | MBD | 9 | No | Small acinar | 6 | LDMT | 523 |
| 13 | Boxer | 11 | Bone | Small acinar | 6 | Piroxicam | N/A |
| 14 | Poodle | 10 | No | Solid | 10 | RP | 213 |
| 15 | Boxer | 13 | Bone, lung | Small acinar | 6 | N/T | 74 |
| 16 | German Shepherd | 12 | Bone, lung | Solid | 10 | LDMT | 375 |
| 17 | MBD | 14 | N/A | Small acinar | 6 | N/A | N/A |
| 18 | MBD | 12 | N/A | Cribriform | 10 | N/T | N/A |
| 19 | MBD | 10 | Lung, liver | Solid | 10 | Piroxicam | 458 |
| 20 | American Pitbull Terrier | 9 | N/A | Cribriform | 10 | N/T | N/A |
| 21 | MBD | 13 | No | Cribriform | 10 | N/A | N/A |
| 22 | Boxer | 11 | Lung | Solid | 10 | LDMT | 101 |
| 23 | Poodle | 14 | No | Small acinar | 6 | LDMT | 432 |
| 24 | MBD | 10 | No | Small acinar | 6 | LDMT | 511 |
| 25 | German Shepherd | 13 | No | Solid | 10 | LDMT | 210 |
| 26 | MBD | 11 | No | Cribriform | 10 | LDMT | 147 |
| PC prostate cancer; MBD Mixed Breed dog; N/A Not Available; N/T No Treatment; RP Radical Prostatectomy; LDMT Low-dose metronomic therapy. * Gleason like score was evaluated according to Palmieri and Grieco, (2015). | | | | | | | |
