# Supplementary material for: Immunohistochemical panel to characterize canine prostate carcinomas according to aberrant p63 expression
Source: PLoS One. 2018 Jun 12;13(6):e0199173. doi: 10.1371/journal.pone.0199173 (PMC5997330; doi:10.1371/journal.pone.0199173)
Supplement: S2 Table — Primary antibodies used for immunohistochemical analysis. (DOCX) [file pone.0199173.s007.docx]

S2 Table. Primary antibodies used for immunohistochemical analysis.

|  |  |  |  |
| --- | --- | --- | --- |
| **Antibody** | **Clone** | **Dilution** | **Manufacturer** |
| P63 | 4A4 | 1:150 |  |
|  |  |  | Dako Cytomation |
| UPIII | AU1 | 1:50 | Progen |
| CK5 | XM26 | 1:50 |  |
|  |  |  | Thermo Scientific |
| HMWC* | 34βE12 | 1:100 |  |
|  |  |  | Dako Cytomation |
| CK8/18 | 5D3 | 1:600 | Leica Biosystems |
| Ki67 | MIB-1 | 1:50 |  |
|  |  |  | Dako Cytomation |
| Chromogranin | Polyclonal | 1:500 |  |
|  |  |  | Abcam |
| CD44 | IM7 | 1:50 |  |
|  |  |  | Santa Cruz |
| CD24 | M1/69 | 1:75 |  |
|  |  |  | Santa Cruz |
| PTEN | MMAC1 | 1:500 |  |
|  |  |  | Bioss |
| AKT | Ser473 | 1:50 |  |
|  |  |  | Cell Signaling |
| C-MYC | C-19 | 1:100 |  |
|  |  |  | Santa Cruz |
| NKX3.1 | P050 | 1:50 | Aviva |
| PSA | Polyclonal | 1:800 | Bioss |
|  |  |  |  |
| AR | ab77557 | 1:100 | Abcam |

*High molecular weight cytokeratin.
